# *Panax notoginseng* Saponins Regulate Macrophage Polarization under Hyperglycemic Condition via NF-*κ*B Signaling Pathway

**DOI:** 10.1155/2018/9239354

**Published:** 2018-07-30

**Authors:** Yan Zhao, Jianlei Zheng, Yongmei Yu, Lihong Wang

**Affiliations:** Department of Cardiology, Zhejiang Provincial People's Hospital, Hangzhou, Zhejiang 310014, China

## Abstract

*Panax notoginseng* saponins (PNS), the principal constituents derived from* Panax notoginseng*, have been extensively used for treating cardiocerebral vascular diseases in China and other Asian countries. The main effects of PNS were anti-inflammatory properties, inhibition of platelet aggregation, improvement of blood flow and insulin resistance, and so on. This study was carried out to explore the effects of PNS on macrophage polarization under hyperglycemic conditions. Human acute monocyte leukemia cell line THP-1 cells were induced into macrophages with Phorbol ester (PMA). Macrophages were then divided into five groups as follows: control (5.5mMol/l glucose), hyperglycemia group (15mMol/l glucose), hyperglycemia plus low-dose PNS (20ug/ml), hyperglycemia plus moderate-dose PNS (40ug/ml), and hyperglycemia plus high-dose PNS (60ug/ml). After 48-hour cell culture, the percentages of M1- and M2-polarized macrophages were measured by flow cytometry analysis. Reverse transcription quantitative real-time polymerase chain reaction (RT-qPCR) was used to evaluate the Ym1 and arginase 1 expression in macrophages. Protein expression of arginase 1, NF-*κ*B p50, p65, and inhibitor of *κ*B (I*κ*B) alpha phosphorylation in macrophages was identified with Western blotting. PNS, especially the high-dose PNS, remarkably increased M2 phenotype ratio in macrophages cultured with hyperglycemia, and the mRNA expression of Ym1 and arginase 1 in macrophages was also upregulated. Meanwhile, PNS remarkably increased the protein expression of arginase 1 and decreased I*κ*B-alpha phosphorylation and subunits of NF-*κ*B p50 and p65 from macrophages in culture medium with hyperglycemia. Taken together, our work demonstrated that PNS promote macrophages to transform M2 phenotype under hyperglycemic conditions through downregulating NF-*κ*B signaling pathway.

## 1. Introduction

Atherosclerosis is regarded as a chronic inflammatory disease, and macrophages play a pivotal role in atherosclerosis from fatty streaks to plaque rupture [1]. Diversity and plasticity are hallmarks for macrophages, and recently substantial evidence demonstrated that plaque pathogenesis and evolution are influenced by macrophage activation and polarization [2]. Macrophages were grossly divided into two main phenotypes, proinflammatory M1 cells (classically activated macrophages) and anti-inflammatory M2 cells (alternatively activated macrophages). Different from phenotypic macrophages with corresponding markers, CD16 is a marker for M1-polarized macrophages, and M2-polarized macrophages are characterized by markers, such as CD206, Ym1, and arginase 1 [3, 4]. M2 phenotype cells are thought to have an important role in atherosclerotic plaques regression [5]. It is mentioned that macrophage polarization is influenced by microenvironment including microbial products and cytokines [6–8]. Hyperglycemia has long been speculated to account for some effects of diabetes on cardiovascular complications caused by atherosclerosis [9]. It is well known that hyperglycemia significantly aggravates inflammatory response and oxidative stress. Importantly, previous studies showed that the phenotype of M1/M2 macrophages was imbalance and macrophages were more inclined to switch to M1 phenotypic polarization under hyperglycemic environment [9, 10].

PNS are the major effective ingredients extracted from* Panax notoginseng*. Numerous studies showed that PNS displayed significant antiatherogenic effects, including the abilities to limit the proliferation of vascular smooth muscle cells, reduce thrombosis risk, protect against artery injury, reduce inflammatory response, and ameliorate hyperglycemia and insulin resistance [11–14]. However, little evidence has been elucidated on the relationship between PNS and macrophage polarization. The present studies were designed to address the effects of PNS on macrophage polarization under hyperglycemic conditions and to explore the relevant molecular mechanism.

## 2. Materials and Methods

### 2.1. Reagents and Chemicals

THP-1 was purchased from Shanghai Institutes for Biological Sciences, Chinese Academy of Sciences (Shanghai, China).* Panax notoginseng* saponins were purchased from Kunming Pharmaceutical Company (KPC) (Yunnan Province, China), which mainly contained notoginsenoside R1 9%, ginsenoside Rg1 32%, ginsenoside Re 5%, ginsenoside Rb1 36%, and ginsenoside Rd 8%. The chemical purity of PNS was about 90%. RPMI-1640 medium without glucose was bought from Gibco (Carlsbad, CA). PMA, D-glucose, and NF-*κ*B inhibitor of BAY 11-7082 were from Sigma-Aldrich (St. Louis, MO, USA). M-MLV Reverse Transcriptase was from GeneCopoeia (Maryland, USA). SYBP Premix Ex Taq TM was from Takara (Shiga, Japan). TRIzol, RIPA, and BCA protein assay kit were from Thermo Fisher Scientific (Waltham, USA). Real-time PCR detection system was from Bio-Rad (Hercules, CA, USA). Flow cytometry was purchased from BD Biosciences (CA, USA). Rabbit polyclonal p50 and p65 antibodies were from Proteintech (Chicago, USA). Mouse monoclonal antibodies to CD16 and CD206 and rabbit monoclonal antibodies to phospho-I*κ*B alpha and arginase 1 were from Abcam (Cambridge, UK). Mouse monoclonal antibody to *β*-actin and rabbit polyclonal antibody to *β*-tubulin were purchased from Boster Biological Technology Co., Ltd. (Wuhan, China) and Cell Signaling Technology (Danvers, MA). ECL kit was purchased from Pierce Biotechnology (Waltham, USA).

### 2.2. Cell Culture and Treatment

The human THP-1 cell, an acute monocytic leukemia cell line, was cultured in RPMI 1640 media supplemented with 10% fetal bovine serum (FBS), 100 units/mL penicillin, and 100ug/ml streptomycin at 37°C in a 5% CO_2_ incubator. THP-1 cells seeded in 6-well culture plates at a density of 5×10^5^ cell/ml with 200nM PMA for 48h were triggered to undergo differentiation into macrophages. Then, nonattached cells were removed by aspiration and adherent cells were washed three times with PBS. Macrophages were then divided into control (5.5mMol/l glucose), hyperglycemic group (15mMol/l glucose), hyperglycemia plus different-dose PNS groups (20ug/ml, 40ug/ml, and 60ug/ml, resp.) and cultured in the corresponding environment for 48h. The THP-1 macrophages were exposed to 5uMol/l NF-*κ*B inhibitor BAY 11-7082 for 2 hours before they were stimulated with hyperglycemia for 24 hours. The experiments were repeated three times independently.

### 2.3. Flow Cytometry

Cultured cells were washed with cold PBS and incubated with CD206 antibody conjugated with PE and CD16 antibody conjugated with FITC at 4°C for 30 min. The cells were centrifuged at 12000 for 2 min at 4°C and resuspended in PBS. The expressions of CD16 and CD206 were determined by flow cytometry to identify the macrophage polarization phenotype with a BD Biosciences Digital LSR II. Anti-CD16 and anti-CD206 antibodies were from Abcam (Cambridge, UK, dilution 1:50 and 1:100, resp.); data were analyzed using FlowJo software (Tree Star, Inc.).

### 2.4. RNA Isolation and Quantitative Real-Time Polymerase Chain Reaction

The total RNA was extracted from macrophages with TRIzol Reagent, and the concentration was measured at 260nm/280nm absorbance ratio. The total RNA was reverse-transcribed to cDNA using a high-capacity cDNA Reverse Transcription Kit. Real-time PCR was carried out with SYBP Premix Ex Taq TM following the manufacturer's instructions. Samples for RT-qPCR test were repeated in triplicate. The PCR was performed on a real-time PCR detection system (Bio-Rad) as follows: 50°C for 2 min and 95°C, 10 min, followed by 40 cycles of denaturation at 95°C for 30 s and annealing at 60°C for 30 s. Semilog amplification curve was analyzed using the 2^−△△Ct^ method, and the relative gene expression was normalized to that of *β*-actin used as an endogenous control. The PCR primer sequences are listed in Table 1.

### 2.5. Protein Extraction and Western Blotting

Macrophages were lysed using Radio-Immunoprecipitation Assay (RIPA) lysis buffer at 4°C for 30 min and subjected to 12000 rpm centrifugation at 4°C for 5 min. The protein concentration was measured by BCA protein assay kit. The lysates were separated by 12% sodium dodecyl sulfate-polyacrylamide gel electrophoresis (SDS-PAGE), transferred to polyvinylidene fluoride (PVDF) membranes, and blocked with 5% skim milk. The blots were analyzed with antibodies according to the manufacturer's instructions and visualized by peroxidase and an enhanced chemiluminescence system. The corresponding antibodies were used in the present study including anti-p50 and anti-p65 (1:1000 dilution and 1:2000 dilution, resp.; Proteintech, USA), anti-arginase 1 and anti-phospho-I*κ*B alpha (1:1000 dilution and 1:10000 dilution, resp.; Cambridge, UK), anti-*β*-actin (1:200 dilution; Boster Biological Technology Co., Ltd., China), and anti-*β*-tubulin (1:1000 dilution; Danvers, MA).

### 2.6. Statistical Analysis

Data analysis was performed with SPSS 13.0 software. Data were expressed as mean ± standard deviation. Comparisons between two groups were evaluated by* t*-test. A* p* value less than 0.05 was considered statistically significant.

## 3. Results

### 3.1. PNS Regulate Macrophage Plasticity towards the M2-Polarized Phenotype under Hyperglycemic Conditions

**Figure 1:**
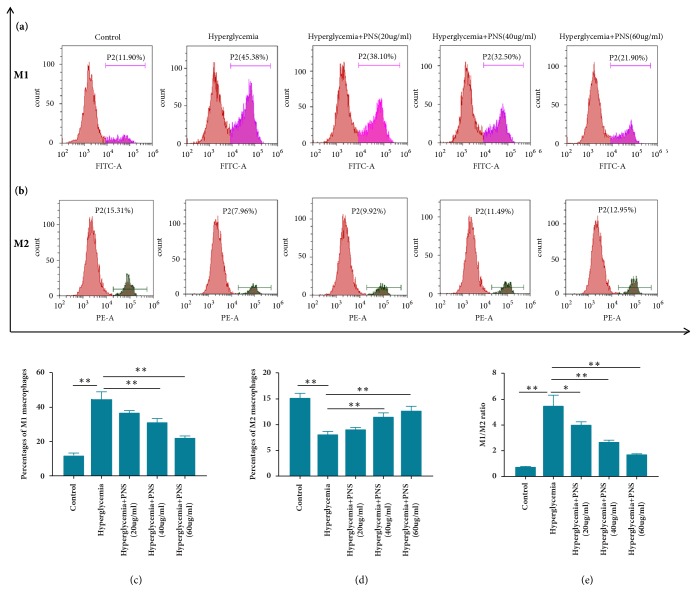
**PNS treatment influences the differentiation of M1 and M2 macrophages under hyperglycemic conditions**. The percentages of M1 and M2 phenotype macrophages under hyperglycemic conditions treated with different levels of PNS were measured according to flow cytometry analysis (a–d). The ratio of M1/M2 phenotype under hyperglycemic conditions treated with different levels of PNS was evaluated (e). CD16 as a biomarker for M1 phenotype macrophages conjugated with FITC and CD206 as a biomarker for M2 phenotype macrophages conjugated with PE, respectively. Data are presented as mean ± SE (n=3 per group). *∗∗* indicates* p*<0.01 and *∗* indicates* p*<0.05.

To explore the correlation between PNS treatment and macrophage polarization, we analyzed the percentages of macrophage phenotype under hyperglycemic conditions treated with different-dose PNS. CD16 and CD206 were used as biological markers for M1 and M2 macrophages, respectively. Our findings indicated that PNS dose-dependently inhibited the M1 macrophage polarization and promoted the macrophages towards M2-polarized phenotype under hyperglycemic conditions after 48-hour culture according to the results of flow cytometry analysis (Figures 1(a)–1(d)). The ratio of M1/M2 decreased with the increasing concentrations of PNS, which was shown in Figure 1(e). These data suggested that PNS may switch the macrophages under hyperglycemic conditions from proinflammatory M1 cells towards an anti-inflammatory M2 phenotype, and the ratio of M1/M2 was changed under hyperglycemic environment with PNS in a dose-independent manner.

### 3.2. The Effect of PNS on Gene Expression of Ym1 and Arginase 1 in Macrophages

**Figure 2:**
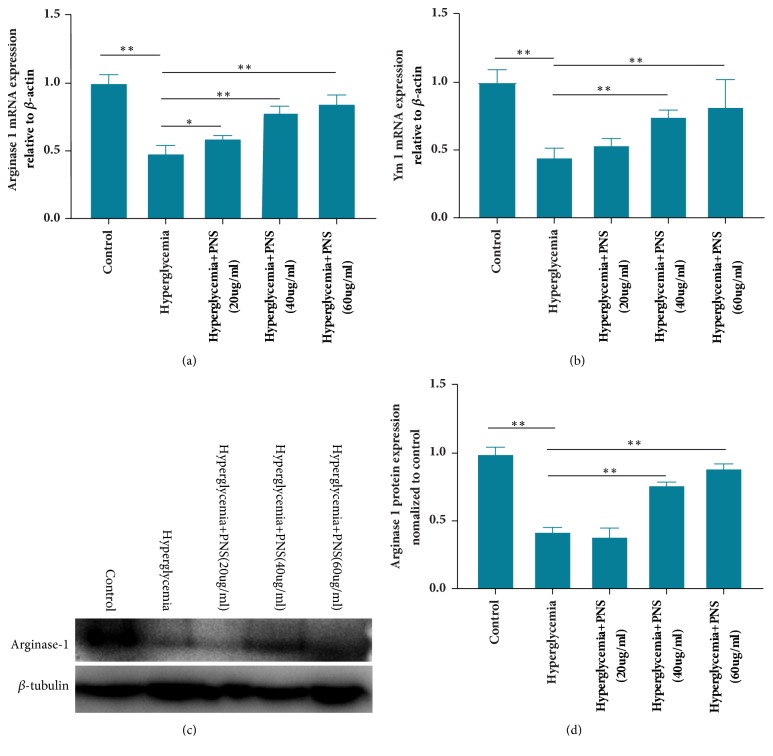
**The effects of PNS treatment on gene expression of Ym1 and arginase I in macrophages under hyperglycemic state.** The effects of different levels of PNS on gene expression of Ym1 and arginase 1 in macrophages under hyperglycemic state were assessed by RT-qPCR (a, b) (n=5 per group). Protein expression of arginase 1 in macrophages under hyperglycemic conditions at different concentration PNS treatment was examined by Western blot (c, d) (n=3 per group). Results are expressed as the mean ± SE. *∗∗* indicates* p*<0.01 and *∗* indicates* p*<0.05.

The gene levels of Ym1 and arginase 1 are important biomarkers for M2 phenotypic macrophages. Real-time PCR results showed that the mRNA expression levels of Ym1 and arginase 1 were lower in hyperglycemic group than in control group (*p*<0.01) (Figures 2(a) and 2(b)). However, these two genes' expression increased in the PNS-treated groups. The levels of Ym1 and arginase 1 in macrophages with moderate- and high-dose PNS treatment were remarkably higher compared with hyperglycemic group (*p*<0.01). The above results demonstrated that PNS regulated macrophage plasticity towards an M2-specified phenotype through influencing the genes expression of Ym1 and arginase 1 under hyperglycemic conditions. We further demonstrated that arginase 1 protein expression was markedly increased under hyperglycemic environment with moderate- and high-dose PNS treatment (Figures 2(c) and 2(d)).

### 3.3. PNS Influence NF-*κ*B Signaling Pathway Regulating M2-Polarized Macrophages Expression

**Figure 3:**
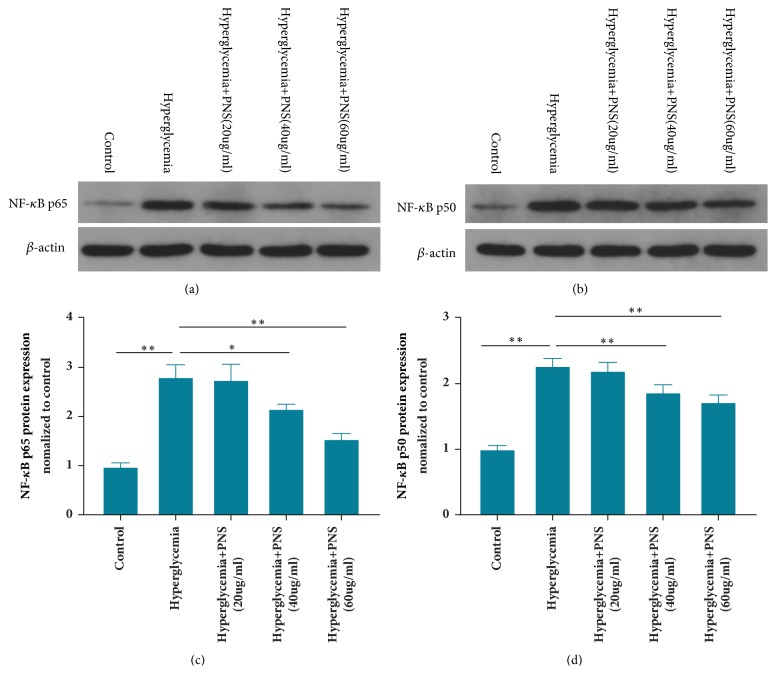
**PNS promote M2-polarized macrophages expression by inhibiting NF-*κ*B signaling pathway.** The protein levels of NF-*κ*B p65 (a, c) and p50 (b, d) in macrophages under hyperglycemic conditions treated with PNS were examined by Western blotting (n=3 per group). Results are expressed as the mean ± SE. *∗∗* indicates* p*<0.01.

**Figure 4:**
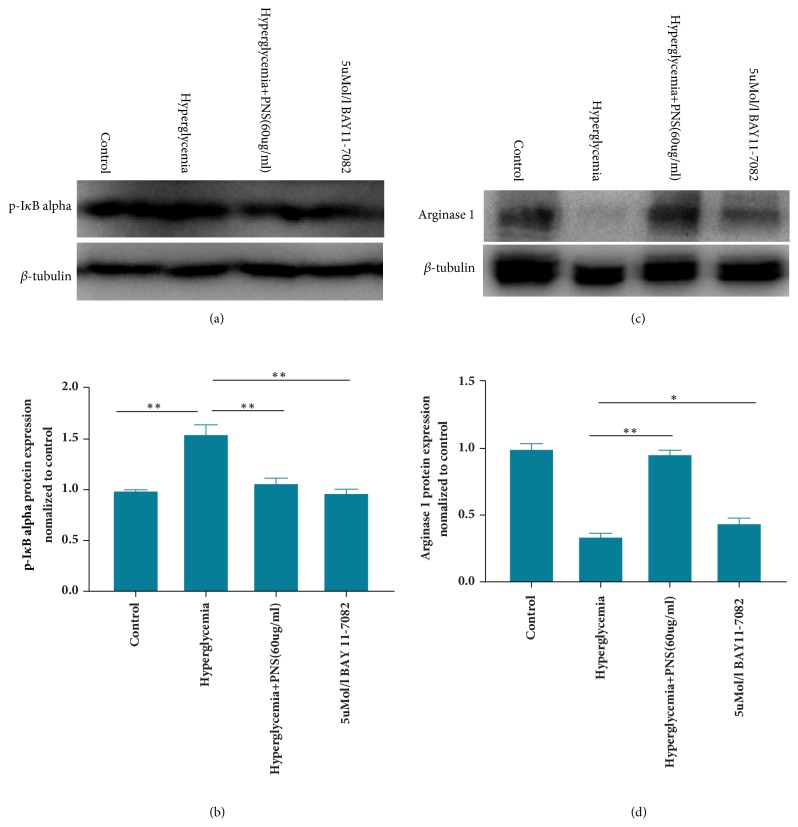
**PNS prompt arginase 1 expression by decreasing I*κ*B-alpha phosphorylation in macrophages under hyperglycemic conditions**. THP-1 macrophages were pretreated with 5uM/l BAY 11-7082 for 2 hours before they were stimulated with 15mMol/l glucose for 24 hours. In this experiment, cell culture time for PNS group is also 24 hours. Effects of 60ug/ml PNS and 5uM/l BAY 11-7082 on I*κ*B-alpha phosphorylation (a, c) and arginase 1 expression (b, d) were determined by Western blot (n=3 per group). Results are expressed as the mean ± SE. *∗*indicates* p*<0.05 and *∗∗* indicates* p*<0.01.

NF-*κ*B signaling pathway plays a pivotal role in inflammation response and macrophage polarization. To explore whether PNS regulate THP-1 derived macrophage polarization through NF-*κ*B signaling pathway under hyperglycemic conditions, subunits p50 and p65 of NF-*κ*B were examined by Western blotting analysis. We found that p50 and p65 expressions in hyperglycemia group were remarkably higher than those in control group (*p*<0.01) (Figure 3). However, the expression of NF-*κ*B p50 and p65 remarkably decreased after moderate- and high-dose PNS treatment (*p*<0.01). Considering the high-dose PNS with the strongest effect on prompting M2 type macrophage polarization and lowering NF-*κ*B subunits p65 and p50 expression, we further tested the effect of high-dose PNS on I*κ*B-alpha phosphorylation. As a result, our study showed that 5uM NF-*κ*B inhibitor BAY 11-7082 and high-dose PNS significantly decreased the expression of I*κ*B-alpha phosphorylation, compared with hyperglycemia group (*p*<0.01) (Figures 4(a) and 4(b)). Meanwhile, the expression of arginase 1 was slightly higher in group with NF-*κ*B inhibitor pretreatment than in hyperglycemia group (*p*<0.05) (Figures 4(c) and 4(d)).

## 4. Discussion

Atherosclerosis is considered as a chronic inflammatory disease, and macrophages play a critical role in the initiation and development of atherosclerosis. In addition to adjusting cholesterol metabolism, forming foam cell, and secreting matrix metalloproteinases and cytokines, it is noted that macrophage polarization under different microenvironment was involved in the pathological process of atherosclerosis [15].* Panax notoginseng* saponins (PNS) are the major effective ingredients extracted from* Panax notoginseng*, which have been extensively used for treating cardiocerebral vascular diseases in China and other Asian countries. In this study, we demonstrated that PNS promoted the macrophages to M2-polarized phenotype under hyperglycemic conditions via downregulating NF-*κ*B signaling pathway.

Plasticity and diversity are hallmarks of macrophages, and a continuum of proinflammatory and anti-inflammatory macrophages mainly including M1 and M2 were known as extreme polarization. M1 and M2 were also called classically and alternatively activated macrophages. Although both M1 and M2 macrophages existed in human atherosclerotic lesions, M1 macrophages are the dominant phenotype linked to plaque progression [16]. The macrophage polarization was a complicated dynamic response to different stimuli, and it is generally accepted that the tissue microenvironment regulates macrophage phenotypic polarization [17].

Cardiovascular diseases are major complications in type 1 and type 2 diabetes. Hyperglycemia may be an important factor associated with cardiovascular complications in diabetic patients [18]. Inflammation and lipid metabolism disorder are more intensive in diabetic patients than in nondiabetic population, which was related to sustained hyperglycemia, aggravating oxygen stress, and formation of advanced glycation end-products (AGEs) [19]. High glucose acts directly or indirectly via the generation of AGEs and reactive oxygen species on endothelial cells, smooth muscle cells, and macrophages [20]. AGE-modified albumin can inhibit SR-B1-mediated efflux of cholesterol to HDL [21]. Hyperglycemic state promotes the macrophages to adhere and transmigrate into the subendothelial space. In addition, hyperglycemia can prevent macrophages from converting into M2 phenotype, and AGEs also play an important role in adjusting the macrophages to form M1-polarized phenotype [9, 10, 22, 23].

Of the various Traditional Chinese Medicines, PNS are one of the most commonly applied products for treating cardiocerebral diseases due to their various beneficial effects. The main pharmacotherapeutic effects of PNS were proven to limit the proliferation of vascular smooth muscle cells, promote blood circulation, improve lipid and glucose metabolism, and inhibit inflammation responses and oxidative stress [11–14, 24, 25]. PNS adjust glucose metabolism by increasing glucose transporter 4 (GLUT4) expression and glycogen synthesis in 3T3-L1 adipocytes [26]. Several studies showed that PNS exert the anti-inflammatory effects on adjusting NF-*κ*B expression, activation of pregnane X receptor, and inhibition of toll-like receptor [13, 27, 28]. However, whether the PNS influence the macrophage polarization is still unknown.

In the current study, we demonstrated that PNS can promote the Ym1 and arginase 1 gene expressions in macrophages under hyperglycemic conditions and in a PNS concentration-dependent manner. It is reported that Ym1 and arginase 1 expression were higher in M2-polarized macrophages than those in M1-polarized macrophages. In the current study, we demonstrated that PNS can promote the Ym1 and arginase 1 gene expression in macrophages under hyperglycemic conditions and in a PNS concentration-dependent manner, which can reflect that PNS induced macrophages to turn into M 2 phenotype. The percentages of M2 phenotype macrophages were increased under hyperglycemic conditions with PNS treatment, which was proven by flow cytometry analysis according to the biomarkers expressed in different polarized macrophages. In addition, we further proved that PNS refrained the production of the NF-*κ*B p50 and p65 subunits and I*κ*B-alpha phosphorylation in macrophages cultured with hyperglycemia. In fact, NF-*κ*B is a key transcription factor involved in various pathological processes such as macrophage polarization [29, 30]. In quiescent cells, NF-*κ*B is mainly located in cytoplasmic environment due to the binding of a dedicated set of inhibitory proteins comprising the I*κ*B family. Upon stimulation, I*κ*B is phosphorylated by activated I*κ*B kinase (IKK). The phosphorylation of I*κ*B causes NF-*κ*B, which can actively shuttle between the nucleus and cytosol, to enter nuclear region and induce gene expression [31]. According to the above findings, we speculated that PNS may promote the expression of M2 type macrophage via decreasing the level of NF-*κ*B signaling pathway mediated by I*κ*B-alpha phosphorylation.

In conclusion, PNS as a Chinese patent medicine for coronary artery disease mainly depend on their effects of anti-inflammation, improving lipid and glucose metabolism. Our work found that anti-inflammation effect of PNS may partly be attributed to the fact that PNS induced macrophages into M2 phenotype, and NF-*κ*B signaling pathway was involved in the macrophage polarization mediated by PNS. However, it is unclear whether the macrophage polarization caused by PNS affects the development of atherosclerosis in vivo. Animal experiments may clarify this mechanism in the following studies.

## Figures and Tables

**Table 1:** Primer sequences for quantitative real-time PCR.

Gene	Primer	Sequences (5′-3′)	Product size
Homo *β*-actin	Forward	5′-AGCGAGCATCCCCCAAAGTT-3′	285bp
	Reverse	5′-GGGCACGAAGGCTCATCATT-3′	
Homo Ym1	Forward	5′-CCTTGACCGCTTCCTCTGTA-3′	166bp
	Reverse	5′-GTTCCATCCTCCGACAGACA-3′	
Homo arginase 1	Forward	5′-TGGACCCATCTTTCACACCA-3′	210bp
	Reverse	5′- GTCCGAAACAAGCCAAGGTT-3′	

## Data Availability

The data used to support the findings of this study are available from the corresponding author upon request.

## Abbreviations

AGEs: Advanced glycation end-products
GLUT4: Glucose transporter 4
I*κ*B: Inhibitor of *κ*B
KPC: Kunming Pharmaceutical Company
NF-*κ*B: Nuclear factor-kappa B
PBS: Phosphate-buffered saline
PNS: * Panax notoginseng* saponins
RT-qPCR: Reverse transcription quantitative real-time polymerase chain reaction
SR-B1: Scavenger receptor class B1
THP-1: Human acute monocyte leukemia cell line.
